# Complex Coronary Instent Chronic Total Occlusion Lesions: Oxidative Stress, Inflammation, and Coronary Stent Lengths

**DOI:** 10.1155/2021/8815048

**Published:** 2021-04-15

**Authors:** Xia Li, Dianxuan Guo, Ying Chen, Youdong Hu, Fenglin Zhang

**Affiliations:** Department of Geriatrics, The Affiliated Huaian Hospital of Xuzhou Medical University, Huaian 223002, China

## Abstract

The oxidative stress and inflammation played the key roles in the development of atherosclerotic coronary plaques. However, the relationships between pro/antioxidant, pro/anti-inflammatory status, and complex coronary instent chronic total occlusion lesions were not clear in the elderly patients with very long stent implantations. We tried to evaluate the roles of pro/antioxidant and pro/anti-inflammatory biomarkers in the diagnosis of complex reocclusion lesions in elderly patients after coronary stenting. We evaluated the expression levels of acrolein (ACR), malondialdehyde (MDA), high sensitivity C-reactive protein (hs-CRP), tumor necrosis factor-*α* (TNF-*α*), superoxide dismutase 3 (SOD3), paraoxonase-1 (PON-1), endothelial nitric oxide synthase (eNOS), and stromal cell-derived factor-1*α* (SDF-1*α*) in the elderly patients with very long stent implantations and complex reocclusion lesions. Levels of ACR, MDA, hs-CRP, and TNF-*α* were remarkably increased (*P* < 0.001), and levels of SOD3, PON-1, eNOS, and SDF-1*α* were decreased significantly (*P* < 0.001) in the elderly patients with very long stents and complex reocclusion lesions. The prooxidant and proinflammatory biomarkers were remarkably increased, as well as antioxidant and anti-inflammatory biomarkers were decreased significantly in the elderly patients with very long stent implantations and complex reocclusion lesions after coronary stenting. In conclusion, these findings indicated that the imbalance between prooxidant/proinflammatory and antioxidant/anti-inflammatory status was associated with complex reocclusion lesions, suggesting that oxidative stress and inflammation played the key roles in progression of complex reocclusion lesions in the elderly patients with very long stent implantations.

## 1. Introduction

The findings show that acrolein (ACR) has the oxidative, proinflammatory, and atherogenic effects. ACR is a key oxidative stress biomarker and leads to the increase in oxidative cellular damage through an inflammatory response, and ACR levels are increased in coronary atherosclerosis and myocardial infarction. ACR elevates the levels of tumor necrosis factor-*α* (TNF-*α*) in endothelial cells and plays a key role in coronary atherosclerosis [1]. The increased malondialdehyde-modified low-density lipoprotein (MDA-LDL) is detected in the plasma of patients with acute coronary syndrome, and MDA-LDL plays an important role in the initiation and progression of coronary atherosclerosis. The increased MDA-LDL is related to endothelial damage and coronary plaque instability in the patients with coronary heart disease [2]. High sensitivity C-reactive protein (hs-CRP) is the important markers of the inflammatory response and proinflammatory trigger, and the inflammation plays the key role in the progression of coronary atherosclerosis. The hs-CRP enhances atherogenic oxidized LDL and is present within lipid-laden coronary plaques, and the hs-CRP in coronary plaque deposition is related to coronary atherosclerosis [3]. The inflammatory response is a key factor in coronary restenosis after primary percutaneous coronary intervention. TNF-*α* is an important proinflammatory cytokine and is the key regulator of inflammatory responses and has a key impact on the progress of coronary restenosis after primary percutaneous coronary intervention [4].

SOD3 is the key source of cells defense against oxidative stress, and the unstability of atherosclerotic plaques is related to a decreased expression of SOD3 [5]. The myocardial expressions of SOD3 that remarkably protect against acute myocardial infarction and elevated cardiac SOD3 levels reduce the myocardial infarct size. The myocardial overexpression of SOD3 inhibits left ventricular remodeling after myocardial infarction, and the SOD3 overexpression has potential clinical utilization in coronary heart disease [6]. Paraoxonase-1 (PON-1) is the antioxidative stress and anti-inflammatory enzymes. The overexpressing human PON-1 decreases oxidative stress and proinflammatory response and inhibits the progression of coronary atherosclerosis by decreasing oxidized LDL in plasma and in the coronary plaque. The decreasing PON-1 activity is an independent cardiovascular risk factor and leads to the elevated oxidative stress and promotes progression of coronary atherosclerosis. Adenovirus-mediated gene transfer of human PON-1 is a potential method to prevent against coronary atherosclerosis in humans [7]. The oxidative stress triggers dysfunction of the endothelium, and the endothelial function is related to the activity and function of endothelial nitric oxide synthase (eNOS). The oxidative stress leads to the reduction of eNOS activity by inhibiting up and downstream signaling pathways of eNOS. The eNOS function and activity of the vascular endothelium are important for maintaining vessel wall integrity and are the novel treatment targets for the cardiovascular diseases [8]. Endothelial progenitor cells are the key participant of vascularization and are mobilized and recruited to the injured vessel wall via signaling pathways with stromal-derived factor (SDF-1*α*). The direct injection of SDF-1*α* to ischemic tissues promotes the accumulation of endothelial progenitor cells and vasculogenesis. The study shows the positive relation between SDF-1*α* levels and endothelial progenitor cells, and SDF-1*α* levels also are demonstrated the inverse association between SDF-1*α* levels and acute vascular lesion [9]. The current study is designed to understand the pro/antioxidant and pro/anti-inflammatory biomarkers in elderly patients with complex reocclusion lesions after different lengths of coronary stent implantation.

## 2. Patients and Methods

### 2.1. Study Population

From 11 October 2010 to 23 July 2018, the present study included 240 consecutive patients with very long stent implantations and complex reocclusion lesions after coronary artery stenting. The inclusion criteria for the patients in the study were (1) patients aged 64 to 85 years old and (2) patients with very long stents and complex chronic instent reocclusion lesions. The present study was approved by the Xuzhou Medical University and the Human Ethics Committee of University Affiliated Huaian Hospital and the Medical Review Board of University in line with the relevant laws and regulations of China, and the patients gave written informed consent in line with the Declaration of Helsinki. The exclusion criteria were (1) Alzheimer's disease, (2) Parkinson's disease, (3) amyotrophic lateral sclerosis, (4) neurodegenerative diseases, (5) the uses of antioxidants and anti-inflammatory drugs, (6) acute coronary syndromes, (7) acute myocardial infarction, (8) acute heart failure, (9) acute renal failure, (10) malignant tumors, (11) allergic reactions to intravascular iodinated contrast medium, (12) acute ischemic stroke, (13) acute or chronic liver diseases, (14) hemodialysis, renal dialysis and peritoneal dialysis, (15) peripheral arterial occlusive disease and venoocclusive disease, and (16) acute and chronic inflammatory diseases.

### 2.2. Research Protocol

The patients without coronary reocclusion were included in without the coronary reocclusion group (*n* = 61). Chronic coronary total reocclusions in the patients after coronary artery implantation were divided into four groups depending on different reocclusion lengths: reocclusion length 4-10 mm group (*n* = 67), reocclusion length 11-20 mm group (*n* = 66), reocclusion length 21-35 mm (*n* = 54), and reocclusion length 36-47 mm (*n* = 53). The different grades of lesion calcifications [10] in the elderly patients with reocclusions after coronary artery implantation were divided into no obvious lesion calcification group (*n* = 58), mild lesion calcification group (*n* = 69), moderate lesion calcification group (*n* = 56), and severe lesion calcification group (*n* = 57). The patients with reocclusions after coronary artery implantation depending on implanted stent lengths were classified into stent length 13-26 mm group (*n* = 57), stent length 30-40 mm group (*n* = 56), stent length 46-56 mm group (*n* = 62), and stent length>56 mm group (*n* = 65). The patients with reocclusions after coronary artery implantation depending on different reocclusion times were divided into coronary reocclusions at 21-25 months group (*n* = 56), coronary reocclusions at 16-20 months group (*n* = 61), coronary reocclusions at 11-15 months group (*n* = 65), and coronary reocclusions at 6-10 months group (*n* = 58).

### 2.3. Quantitative Analysis of Coronary Angiographies and Clinical Ultrasound Images

Coronary artery chronic total reocclusions were evaluated by the quantitative coronary angiographic system (QAngio XA MEDIS, Medical Imaging System BV, Leiden, the Netherlands). The coronary artery angiographies were examined by two blinded senior interventional cardiologists. The clinical ultrasound images were examined with synchronized electrocardiography recordings by the two expert cardiologists blinded to patients' clinical information according to American Society of Echocardiography guidelines.

### 2.4. Levels of ACR, MDA, hs-CRP, and TNF-*α*

The blood amino acids were completely eliminated by the chromatography on cellulose phosphate before rapid analysis of polyamines, and the contents of polyamines were examined by the high-performance liquid chromatography method as it was described. The ACR in the blood was formed from 3-aminopropanal and was examined as it was described [11]. The blood samples for MDA evaluation were immediately stored at -70°C until they were assayed. MDA levels in human blood were measured spectrophotometrically at 535 nm, and the descriptive results were finally reported as nmol/L [12]. The peripheral venous blood samples were collected for the determination of hs-CRP levels, and the blood samples for measuring hs-CRP were stored at -80°C until the samples were examined. The blood levels of hs-CRP were determined using hs-CRP enzyme-linked immunosorbent assay kit from Roche Diagnostics GmbH, Mannheim, Germany [13]. The blood samples for TNF-*α* evaluation were collected, and the samples were centrifuged for seven minutes in quality centrifuge with the motor cooling system, and the serum samples were then stored at -80°C until the samples were examined. The serum TNF-*α* was determined using a enzyme-linked immunosorbent assay method (enzyme-amplified sensitivity immunoassay kits purchased from R&D Systems INC., Minneapolis, MN, USA), and the TNF-*α* levels were finally reported as ng/L [14].

### 2.5. Levels of SOD3, PON-1, eNOS, and SDF-1*α*

The blood samples were centrifuged at 3200 rpm for 25 minutes, and the SOD3 activity in plasma was examined with enzyme-linked immunosorbent assay kit in line with the manufacturer's operating instructions. The SOD3 activity assay was performed with the commercial RANSOD-Randox, UK. The assay data were finally reported as U/mL [15]. Enzyme activity of PON-1 using paraoxon and phenyl acetate as respective substrate was estimated as it was described. The rate of hydrolysis of paraoxon substrate (the buffer containing 5.5 mM in 100 mM Tris/HCL and 1 mM CaCl2 pH 8.0) was determined spectrophotometrically at 415 nm by measuring the absorbances, and the enzyme activity of PON-1 was reported as U/L [16].

The levels of eNOS were reported as pg/mL, and the detection range was from 0.156 to 1000 pg/mL. All EDTA-anticoagulated plasma samples were taken from each patient after the overnight fast. Blood samples from the research central laboratory were centrifuged immediately at 1500 rpm for 15 min at 4°C and stored at -80°C until the samples were examined. All samples were duplicately evaluated as well as the coefficient of intra-assay variation was 3.5% for the intra-assay test. The eNOS concentrations were examined by Sigma's Sandwich enzyme-linked immunosorbent assay kit [17]. The levels of SDF-1*α* were examined in duplicate with monoclonal antibody-based enzyme immunoassays (Quantikine, enzyme immunoassays kit, R&D Systems, Minneapolis, MN, USA). The levels of SDF-1*α* were evaluated in platelet-poor plasma after centrifugation at 1000 g for 10 min at 4°C. The results were finally reported as ng/L [9].

### 2.6. Data Analysis

The results of these analyses were presented as the mean ± standard deviations (mean ± SD). The paired Student's *t*-tests, paired test, were used to determine each pair of the matching observed clinical data of the patients, and the one-way analysis of variance (ANOVA) was employed to examine the differences among the means. *P* values less than 0.05 were considered to be statistically significant for the results. All statistical analyses of the data were done by using the SPSS statistics for windows version 20.0 software (SPSS: An IBM Company, IBM Corporation, Armonk, NY, USA) in evaluating the clinical utility of biomarkers.

## 3. Results

### 3.1. Baseline Characteristics of the Elderly Patients with Chronic Instent Reocclusion Lesions after Coronary Stent Placement

The baseline patient characteristics were very similar within each study group of the elderly patients (Table 1). The elderly patients in every group were well matched without statistically significant differences in age, gender, marital status, target lesion revascularization, body weight, body height, type II diabetes, coronary heart disease, hypertension, myocardial infarction, angina pectoris, dyslipidaemia, obesity, and the duration of illness.

### 3.2. Biomarker Levels Related to Different Reocclusion Lengths in the Elderly Patients with Very Long Stent Implantations

The levels of ACR, MDA, hs-CRP, and TNF-*α* were increased significantly in the reocclusion length 21-35 mm group when compared with the reocclusion length 11-20 mm group and reocclusion length 4-10 mm group, respectively (*P* < 0.001) and were further increased significantly in the reocclusion length 36-47 mm group compared to the reocclusion length 21-35 mm group and reocclusion length 11-20 mm group, respectively (*P* < 0.001). The levels of SOD3, PON-1, eNOS, and SDF-1*α* were reduced remarkably in the reocclusion length 21-35 mm group when compared with the reocclusion length 11-20 mm group and reocclusion length 4-10 mm group, respectively (*P* < 0.001) and were further reduced remarkably in the reocclusion length 36-47 mm group compared to the reocclusion length 21-35 mm group and reocclusion length 11-20 mm group, respectively (*P* < 0.001). These findings suggested that high levels of ACR, MDA, hs-CRP, and TNF-*α* and low levels of SOD3, PON-1, eNOS, and SDF-1*α* were associated with reocclusion lengths in the elderly patients with very long stent implantations. The coronary reocclusion lesion lengths were related to pro/antioxidant and pro/anti-inflammatory imbalances in the elderly patients with very long stent implantations (Table 2).

### 3.3. Biomarkers Related to Different Grades of Reocclusion Lesion Calcifications in the Elderly Patients with Very Long Stent Implantations

The levels of ACR, MDA, hs-CRP, and TNF-*α* were increased significantly in the moderate lesion calcification group when compared with the mild lesion calcification group and no obvious lesion calcification group, respectively (*P* < 0.001) and were further increased significantly in the severe lesion calcification group compared to the moderate lesion calcification group and mild lesion calcification group, respectively (*P* < 0.001). The levels of SOD3, PON-1, eNOS, and SDF-1*α* were reduced remarkably in the moderate lesion calcification group when compared with the mild lesion calcification group and no obvious lesion calcification group, respectively (*P* < 0.001) and were further reduced remarkably in the severe lesion calcification group compared to the moderate lesion calcification group and mild lesion calcification group, respectively (*P* < 0.001). The results showed that increased levels of ACR, MDA, hs-CRP, and TNF-*α* and decreased levels of SOD3, PON-1, eNOS, and SDF-1*α* were associated with different grades of reocclusion lesion calcifications in the elderly patients with very long stent implantations. The pro/antioxidant and pro/anti-inflammatory imbalances may contribute to severe reocclusion lesion calcifications in the elderly patients with very long stent implantations (Table 3).

### 3.4. Biomarker Levels and Different Stent Lengths in the Elderly Patients after Coronary Artery Implantation

Levels of ACR, MDA, hs-CRP, and TNF-*α* were increased significantly in the stent length 46-56 mm group compared to the stent length 30-40 mm group and stent length 13-26 mm group, respectively (*P* < 0.001) and were further increased significantly in the stent length > 56 mm group compared to the stent length 46-56 mm group and stent length 30-40 mm group, respectively (*P* < 0.001). The levels of SOD3, PON-1, eNOS, and SDF-1*α* were reduced remarkably in the stent length 46-56 mm group when compared with the stent length 30-40 mm group and stent length 13-26 mm group, respectively (*P* < 0.001) and were further reduced remarkably in the stent length > 56 mm group compared to the stent length 46-56 mm group and stent length 30-40 mm group, respectively (*P* < 0.001). The biomarker levels of pro/antioxidant and pro/anti-inflammatory imbalances depended on stents of different lengths in the elderly patients after coronary artery implantation. The very long stents promoted the pro/antioxidant and pro/anti-inflammatory imbalances and may have a significantly higher risk of developing complex reocclusion lesions after coronary artery implantation (Table 4).

### 3.5. Biomarker Levels and Different Reocclusion Times in the Elderly Patients with Very Long Stents

Levels of ACR, MDA, hs-CRP, and TNF-*α* were increased significantly in coronary reocclusions at the 11-15 month group compared to coronary reocclusions at the 16-20 month group and coronary reocclusions at the 21-25 month group, respectively (*P* < 0.001) and were further increased significantly in coronary reocclusions at the 6-10 month group compared to coronary reocclusions at the 11-15 month group and coronary reocclusions at the 16-20 month group, respectively (*P* < 0.001). The levels of SOD3, PON-1, eNOS, and SDF-1*α* were reduced remarkably in coronary reocclusions at the 11-15 month group when compared with coronary reocclusions at the 16-20 month group and coronary reocclusions at the 21-25 month group, respectively (*P* < 0.001) and were further reduced remarkably in coronary reocclusions at the 6-10 month group compared to coronary reocclusions at the 11-15 month group and coronary reocclusions at the 16-20 month group, respectively (*P* < 0.001). The results showed that the pro/antioxidant and pro/anti-inflammatory imbalances may further accelerate the formation of complex reocclusion lesions in the elderly patients with very long stents (Table 5).

### 3.6. ACR, MDA, Hs-CRP, TNF-*α*, SOD3, PON-1, eNOS, and SDF-1*α* as Independent Indicators of Complex Reocclusion Lesions after Different Lengths of Coronary Stent Implantation

By using the multiple regression analysis, ACR, MDA, hs-CRP, TNF-*α*, SOD3, PON-1, eNOS, and SDF-1*α* were found to be related to independent risk indicators of complex reocclusion lesions following different lengths of coronary stent implantation after adjustment for age, gender, type II diabetes, coronary heart disease, hypertension, myocardial infarction, angina pectoris, dyslipidaemia, married, unmarried, obesity, and target lesion revascularization in the patients complex reocclusion lesions. The *P* value of <0.05 was considered to indicate a statistically significant difference (Table 6).

## 4. Discussion

The coronary stent implantation increases oxidative stress, and oxidation sensitive signaling pathways play an important pathogenetic role in development of artery instent restenosis. Therapy against oxidative stress is the promising strategy for treatment of artery stent-induced restenosis [18]. The clinical researches demonstrate that the inflammatory response plays a key role in the artery neointimal hyperplasia and coronary instent restenosis in the patients with coronary heart disease after coronary stent implantation [19].

The oxidative modifications of myocardial protein by ACR are related to cardiac mitochondrial oxidative stress and atherosclerosis. The endothelium of blood vessels is a vulnerable target for ACR and the exposure to ACR results in endothelial injury. ACR elevates the levels of TNF-*α* in endothelial cells, and the proinflammatory and proatherogenic effects of ACR contribute to blood vessel endothelial injury and inflammatory response. ACR plays a crucial role in the progression of atherosclerosis through the inflammatory response and oxidative stress [1, 20]. MDA-LDL as an oxidative stress form is found in atherosclerotic lesions and plasma. The oxidative modification of LDL is a key factor in progression of coronary atheroscrelosis. MDA-LDL is associated with coronary plaque vulnerability in patients with coronary heart disease and is an independent predictor for the coronary plaque development in the patients after percutaneous coronary intervention. Oxidized LDL also leads to the proinflammatory response and induces endothelial injury. The elevated level of oxidized LDL plays an important role in formation of unstable atherosclerotic plaque and is the diagnostic marker of coronary plaque vulnerability and thin-cap fibroatheroma [2].

Inflammation plays a key role in the progression of atherosclerotic plaques. hs-CRP is the biomarkers of the proinflammatory response and cardiovascular risk. There is a significant relationship between hs-CRP and atherogenic oxidized LDL in coronary plaque, and hs-CRP promotes coronary atherosclerosis. Circulating levels of hs-CRP are shown to be elevated in the patients with acute myocardial infarction and chronic heart failure [21]. TNF-*α* induces oxidative stress, and TNF-*α*-blocking agents have an effect on oxidative stress [22]. The proinflammatory cytokine TNF-*α* is involved in the process of coronary restenosis after percutaneous coronary intervention in humans. The studies report that the lack of the TNF-*α* gene expression inhibits the narrowing of vascular lumena [4].

Our study showed that the levels of ACR, MDA, hs-CRP, and TNF-*α* as biomarkers of oxidative stress and inflammation were increased significantly in the elderly patients with very long stents and complex reocclusion lesions, and the levels of ACR, MDA, hs-CRP, and TNF-*α* were increased remarkably in coronary reocclusions at 6-10 months when compared with coronary reocclusions at 11-25 months after very long stent implantations. The study shows that the long stent implantations increase oxidative stress and inflammatory response. The oxidative stress and inflammatory response play the important roles in development of neointimal hyperplasia and instent restenosis in the patients after coronary stent implantations [18, 19]. ACR plays a crucial role in the progression of atherosclerosis through the inflammatory response and oxidative stress [1, 20]. The elevated level of MDA-LDL is associated with coronary plaque vulnerability in the patients after percutaneous coronary intervention [2]. hs-CRP is the biomarker of the proinflammatory response and plays a key role in the progression of coronary atherosclerotic plaques [21]. TNF-*α* induces the proinflammatory response and oxidative stress [22], and the TNF-*α* gene expressions promote the coronary luminal stenosis after percutaneous coronary intervention in humans [4]. Our results suggested that the levels of ACR, MDA, hs-CRP, and TNF-*α* as biomarkers of oxidative stress and inflammation were associated with very long stent implantations and complex reocclusion lesions. The long stent implantations may contribute to oxidative stress and inflammation, and the interplay of oxidative stress and inflammation may accelerate the formation of complex chronic total reocclusions in the elderly patients after very long stent implantations.

The proinflammatory response and prooxidative stress are the critical pathological factors for atherosclerotic plaque instability in humans. SOD3 has an anti-inflammatory role in ischemic tissue injuries, and SOD3 decreases inflammatory response in vascular lesions. Overexpressions of SOD3 remarkably low TNF-*α* levels in ischemic tissue injury and SOD3 are a possible candidate for the therapy of inflammatory disorders [23]. SOD3 is the important defense enzyme against the oxidative stress and artery atherosclerotic lesions. The instability of atherosclerotic plaques is related to decreased expressions of SOD3, and the expressions of SOD3 protect the heart against both myocardial infarction and cardiac remodeling, and the increased cardiac SOD3 levels are shown to decrease the myocardial infarct size [5, 6]. Human PON-1 is the high density lipoprotein-associated enzyme that inhibits proinflammatory lipids formed from the oxidized LDL. The adenovirus-mediated expressions of human PON-1 remarkably reduce the progress of atherosclerosis, artery plaque volume, oxidized LDL levels, and oxidative stress in the coronary plaques and plasma. The use of adenovirus expressions of human PON-1 may be a useful method for preventing coronary atherosclerosis in humans [7].

Oxidative stress promotes the endothelial eNOS dysfunction via uncoupling in coronary arteries and the procession of atherosclerosis. The elevated oxidative stress in artery endothelial cells leads to eNOS uncoupling, and antioxidant therapy inhibits eNOS uncoupling and improves artery endothelial function. Inflammation is closely related to oxidative stress and promotes the endothelial dysfunction and development of cardiovascular disease [8]. Blood vessel endothelium injury and dysfunction promote the procession of coronary atherosclerosis, coronary artery disease, and coronary restenosis after percutaneous coronary intervention. Although coronary stents are coated with antirestenosis drugs to inhibit the overproliferation and migration of coronary smooth muscle cells, the agents also slow reendothelialization of arteries and promote coronary restenosis. eNOS gene transfer improves heart repair after myocardial infarction, and the eNOS overexpression reduces neointimal hyperplasia and coronary restenosis [24]. SDF-1*α* has the anti-inflammatory effect of TNF-*α* and plays a key role in coronary heart disease. The SDF-1*α* levels are remarkably reduced in the patients with stable and unstable angina pectoris. SDF-1*α* inhibits the inflammatory mediators and promotes coronary plaque stabilization. The treatment intervention that increased activity of SDF-1*α* would be potentially beneficial for atherosclerosis and acute coronary syndromes [25]. SDF-1*α* also decreases oxidative stress and has the cytoprotective effects against oxidative stress injuries that could be an innovative method for clinical use [26].

Our findings showed that the levels of SOD3, PON-1, eNOS, and SDF-1*α* as biomarkers of antioxidative stress and anti-inflammation were decreased significantly in the elderly patients with very long stent implantations and complex reocclusion lesions, and the levels of SOD3, PON-1, eNOS, and SDF-1*α* were decreased remarkably in coronary reocclusions at 6-10 months when compared with coronary reocclusions at 11-25 months after very long stent implantations. The proinflammatory response and prooxidative stress are the critical pathological factors for atherosclerotic plaque instability in humans. SOD3 is the important defense enzyme against the oxidative stress and inflammation in artery atherosclerotic lesions. The instability of atherosclerotic plaques is related to decreased expressions of SOD3, and the increased cardiac SOD3 levels decrease the myocardial infarct size [5, 6, 23]. The adenovirus-mediated expressions of human PON-1 remarkably reduce the oxidative stress and inflammation in the coronary plaques and prevent the progression of coronary atherosclerosis. The use of adenovirus expressions of human PON-1 may be a useful method for inhibiting coronary atherosclerosis in humans [27]. Inflammation is closely related to oxidative stress and promotes the endothelial dysfunction and development of cardiovascular disease. eNOS inhibits vascular inflammation and development of vasculitic lesion formation, and the eNOS overexpression reduces neointimal hyperplasia and coronary restenosis. Oxidative stress promotes the endothelial eNOS dysfunction via uncoupling in coronary arteries and the procession of atherosclerosis [8, 24, 27, 28]. The present study suggested that the crossactivation of proinflammation and prooxidative stress and the imbalances of pro/antioxidant and pro/anti-inflammatory mechanisms may accelerate the formation of complex instent reocclusion lesions in the elderly patients after very long stent implantations.

The results of the present study showed that levels of ACR, MDA, hs-CRP, and TNF-*α* were increased in elderly patients with complex reocclusion lesions after coronary stenting. ACR as marker of oxidative stress is the convergence of pathogenic signaling pathways of oxidative stress, and the majority of oxidative stress is manifested by ACR, and ACR has a long half-life and is an important oxidative stress-related molecule [29, 30]. MDA production is formed after the breakdown of lipid hydroperoxides and is a biomarker of free radical injury in pathologies associated with free radical and oxidative stress. The levels of MDA as a marker of the oxidative stress response are considered as a sign of cell injury and promote the oxidant/antioxidant imbalance in cells [31, 32]. hs-CRP as an inflammatory marker promotes acute and chronic inflammatory responses and is related to an adverse prognosis. High levels of hs-CRP are associated with coronary artery calcification and myocardial oxidative stress. Patients with myocardial cell damage have more severe systemic inflammatory response [33, 34]. The proinflammatory cytokine TNF-*α* is the key cell signaling pathway and potent proinflammatory mediator involved in the inflammatory response. The proinflammatory cytokine TNF-*α* promotes the inflammatory response through activating the nuclear factor kappa-light-chain-enhancer of activated *β* cells. The high levels of proinflammatory cytokine TNF-*α* are considered as indicative of the pathological injury. The overexpression of proinflammatory cytokine TNF-*α* is related to adverse pathological and clinical outcomes [35, 36]. Our data indicated that the upregulation of ACR, MDA, hs-CRP, and TNF-*α* as prooxidant and proinflammatory biomarkers may play a key role in complex reocclusion lesions in elderly patients after coronary stenting.

Antioxidant defense enzyme SOD3 in cells prevents the oxidative stress response. Prooxidative and antioxidative imbalance promotes upregulation of oxidative stress levels, and an increase in oxidative stress is capable of producing harmful effects of oxidative stress. SOD is a cellular antioxidant defense enzyme for oxidative attacks [37]. Oxidative stress is closely related to the pathological mechanisms of different chronic diseases. The expression of the antioxidant enzyme SOD protects the cells from oxidative stress-associated damage. High levels of MDA reduce antioxidant enzyme levels of SOD and total antioxidant ability [38]. Oxidative stress plays a key role in PON-1 inactivation and decreased activity of PON-1 as an anti-oxidative stress biomarker impairs function of high-density lipoprotein cholesterol, as the cholesterol carrier, which inhibits antioxidative and anti-inflammatory function [39]. PON-1 exerts potent antioxidative and anti-atherogenic effects and inhibits the accumulation of oxidized low-density lipoprotein and the formation of the atherosclerotic plaques. Decreased PON-1 activity increases proinflammatory and proatherogenic responses and is related to risk factor for coronary artery disease [40]. The eNOS has anti-oxidative stress and anti-inflammation functions and inhibits oxidative stress-related damages and inflammatory responses. High levels of oxidative stress promote the uncoupling of eNOS, directly leading to oxidative stress damages in vascular function and the progression of cardiometabolic disease [41, 42]. SDF-1*α* alleviates oxidative stress damage by inhibition of the mitochondrial oxidative stress response and inhibits inflammatory responses. Upregulating the SDF-1*α* expression totally inhibits the enhanced oxidative stress by decreasing oxidized low0density lipoprotein and improves antioxidant capacity. Upregulating SDF-1*α* significantly enhances nuclear accumulation of nuclear factor E2-related factor 2 (Nrf2) and the expressions of antioxidative stress response genes [43–45]. The current data demonstrated the downregulation of SOD3, PON-1, eNOS, and SDF-1*α* as antioxidant and anti-inflammatory biomarkers that were related to complex reocclusion lesions in elderly patients after coronary stenting.

Percutaneous coronary intervention (PCI) induces coronary injury on the endothelial cells which is related to the proinflammatory response and oxidative stress. PCI increases the levels of the inflammatory response and pathophysiological levels of oxidative stress response, which promotes the progression of coronary restenosis in patients with coronary artery disease after PCI [46, 47]. Coronary atherosclerotic lesion formation is related to oxidative stress and inflammatory response. The formation of coronary atherosclerotic plaques is accelerated by the interaction between oxidative stress and inflammatory response [48]. The coronary arteries have the oxidant/antioxidant system, and high levels of oxidative stress induced by the imbalance of oxidative stress/antioxidative stress play a crucial role in the progression of coronary atherosclerotic lesions. Oxidative stress also increases the proinflammatory response, and the proinflammatory process induces more severe oxidative stress. Interplay between oxidative stress and inflammatory response promotes coronary endothelial injury and formation of coronary atherosclerotic plaques [48, 49]. In our study, we observed the effects of pro/antioxidant and pro/anti-inflammatory biomarker changes on complex reocclusion lesions in elderly patients after coronary stenting. Recent studies have demonstrated that the imbalance of the oxidative/antioxidation system and the interaction between oxidative stress and inflammatory response accelerate progression of coronary atherosclerotic lesions [48, 49].

## 5. Conclusion

The present study showed that the levels of pro/antioxidant and pro/anti-inflammatory markers were related to instent reocclusion lesions. The crossactivation of proinflammation and prooxidative stress and pro/antioxidant and pro/anti-inflammatory imbalances may further accelerate the formation of complex instent reocclusion lesions. Pro/antioxidant and pro/anti-inflammatory biomarkers may have therapeutic and prognostic significance in elderly patients with complex reocclusion lesions after different lengths of coronary stent implantation.

## Figures and Tables

**Table 1:** Baseline characteristics of the elderly patients with coronary reocclusions after coronary artery stent implantation.

	Without coronary reocclusion *n* = 61	Right coronary reocclusion *n* = 57	Left main coronary reocclusion *n* = 59	Left circumflex coronary reocclusion *n* = 64	Left anterior descending coronary reocclusion *n* = 60
Age (years)	62.5 ± 3.0	67.3 ± 4.0	71.4 ± 4.6	74.9 ± 4.2	80.4 ± 4.9
Gender [male/female (M/F)]	29/32	31/26	23/16	30/34	39/33
Type II diabetes (M/F)	18/21	20/15	16/19	25/16	29/21
Coronary heart disease (M/F)	34/27	31/26	19/20	34/30	35/37
Hypertension (M/F)	24/17	19/23	13/18	29/17	24/30
Myocardial infarction (M/F)	31/30	27/30	20/19	34/30	33/39
Angina pectoris (M/F)	29/18	14/25	17/13	32/21	27/23
Coronary angiographies (M/F)	36/25	26/31	28/11	37/27	35/37
Echocardiographic assessments (M/F)	36/25	26/31	28/11	37/27	35/37
Dyslipidaemia (M/F)	27/20	17/23	14/21	26/18	30/19
Married (M/F)	29/20	20/17	16/15	24/23	25/31
Unmarried (M/F)	5/7	11/9	5/3	10/7	7/9
Obesity (M/F)	21/10	30/15	18/13	26/19	30/21
Drug-eluting stents (M/F)	37/24	27/30	23/16	30/34	32/40
Target lesion revascularization (M/F)	37/24	27/30	23/16	30/34	32/40

**Table 2:** Biomarkers related to different reocclusion lengths in the elderly patients with very long stent implantations.

	Without coronary reocclusion *n* = 61	Reocclusion length 4-10 mm *n* = 67	Reocclusion length 11-20 mm *n* = 66	Reocclusion length 21-35 mm *n* = 54	Reocclusion length 36-47 mm *n* = 53
ACR (nmol/mL)	0.9 ± 0.2	1.4 ± 0.9^∗^	3.9 ± 1.4^∗∗^	7.0 ± 3.2^∗∗∗^	11.4 ± 4.5^∗∗∗∗^
MDA (nmol/L)	3.5 ± 1.4	4.9 ± 1.7^∗^	6.7 ± 2.9^∗∗^	8.5 ± 3.7^∗∗∗^	12.0 ± 4.9^∗∗∗∗^
hs-CRP (mg/L)	2.7 ± 0.8	3.9 ± 0.9^∗^	6.1 ± 1.3^∗∗^	10.2 ± 3.0^∗∗∗^	14.9 ± 4.2^∗∗∗∗^
TNF-*α* (ng/L)	20.1 ± 6.2	31.0 ± 6.9^∗^	51.3 ± 7.6^∗∗^	62.9 ± 7.9^∗∗∗^	90.8 ± 8.2^∗∗∗∗^
SOD3 (U/mL)	17.0 ± 3.0	15.1 ± 2.6^∗^	12.9 ± 1.7^∗∗^	9.9 ± 0.8^∗∗∗^	5.0 ± 0.6^∗∗∗∗^
PON-1 (U/L)	157.4 ± 63.5	130.7 ± 59.2^∗^	107.0 ± 50.1^∗∗^	87.9 ± 45.6^∗∗∗^	40.2 ± 37.2^∗∗∗∗^
eNOS (pg/mL)	59.7 ± 23.5	50.5 ± 20.9^∗^	32.9 ± 18.7^∗∗^	22.8 ± 11.0^∗∗∗^	18.9 ± 9.0^∗∗∗∗^
SDF-1*α* (ng/L)	18.1 ± 4.3	17.9 ± 4.0^∗^	14.9 ± 3.2^∗∗^	10.0 ± 2.6^∗∗∗^	7.1 ± 1.2^∗∗∗∗^

^∗^ *P* < 0.001 (without coronary reocclusion group/reocclusion length 4-10 mm group). ^∗∗^*P* < 0.001 (reocclusion length 4-10 mm group/reocclusion length 11-20 mm group). ^∗∗∗^*P* < 0.001 (reocclusion length 11-20 mm group/reocclusion length 21-35 mm group). ^∗∗∗∗^*P* < 0.001 (reocclusion length 21-35 mm group/reocclusion length 36-47 mm group).

**Table 3:** Biomarkers related to different grades of reocclusion lesion calcifications in the elderly patients with very long stent implantations.

	Without coronary reocclusion *n* = 61	No obvious lesion calcifications *n* = 58	Mild lesion calcifications *n* = 69	Moderate lesion calcifications *n* = 56	Severe lesion calcifications *n* = 57
ACR (nmol/mL)	0.9 ± 0.2	0.8 ± 0.3	2.1 ± 0.7^∗^	5.0 ± 2.0^∗∗^	6.9 ± 3.5^∗∗∗^
MDA (nmol/L)	3.5 ± 1.4	3.9 ± 1.2	7.0 ± 2.0^∗^	12.8 ± 3.9^∗∗^	17.0 ± 5.2^∗∗∗^
hs-CRP (mg/L)	2.7 ± 0.8	2.9 ± 0.4	4.6 ± 0.6^∗^	6.9 ± 0.8^∗∗^	10.2 ± 3.2^∗∗∗^
TNF-*α* (ng/L)	20.1 ± 6.2	18.9 ± 6.3	21.4 ± 7.2^∗^	25.9 ± 8.0^∗∗^	30.5 ± 10.2^∗∗∗^
SOD3 (U/mL)	17.0 ± 3.0	16.9 ± 3.1	14.1 ± 2.7^∗^	10.3 ± 2.0^∗∗^	6.1 ± 1.7^∗∗∗^
PON-1 (U/L)	157.4 ± 63.5	158.0 ± 62.5	124.9 ± 60.0^∗^	90.2 ± 57.3^∗∗^	46.4 ± 30.9^∗∗∗^
eNOS (pg/mL)	59.7 ± 23.5	60.3 ± 21.6	43.7 ± 27.0^∗^	35.8 ± 25.8^∗∗^	19.0 ± 17.5^∗∗∗^
SDF-1*α* (ng/L)	18.1 ± 4.3	17.5 ± 3.1	15.1 ± 2.7^∗^	10.8 ± 1.9^∗∗^	7.6 ± 1.1^∗∗∗^

^∗^ *P* < 0.001 (no obvious lesion calcification group/mild lesion calcification group). ^∗∗^*P* < 0.001 (mild lesion calcification group/moderate lesion calcification group). ^∗∗∗^*P* < 0.001 (moderate lesion calcification group/severe lesion calcification group).

**Table 4:** Biomarker levels and different stent lengths in the elderly patients coronary artery stent implantations.

	Stent length < 12 mm *n* = 61	Stent length 13-26 mm *n* = 57	Stent length 30-40 mm *n* = 56	Stent length 46-56 mm *n* = 62	Stent length > 56 mm *n* = 65
ACR (nmol/mL)	0.9 ± 0.2	2.1 ± 0.4^∗^	5.7 ± 0.9^∗∗^	10.0 ± 1.3^∗∗∗^	12.8 ± 1.7^∗∗∗∗^
MDA (nmol/L)	3.5 ± 1.4	4.9 ± 1.6^∗^	7.1 ± 1.9^∗∗^	12.0 ± 2.4^∗∗∗^	14.9 ± 3.0^∗∗∗∗^
hs-CRP (mg/L)	2.7 ± 0.8	3.0 ± 0.9^∗^	6.5 ± 1.8^∗∗^	8.9 ± 2.1^∗∗∗^	13.5 ± 3.6^∗∗∗∗^
TNF-*α* (ng/L)	20.1 ± 6.2	23.1 ± 6.9^∗^	27.9 ± 7.1^∗∗^	39.3 ± 8.0^∗∗∗^	54.1 ± 9.4^∗∗∗∗^
SOD3 (U/mL)	17.0 ± 3.0	15.2 ± 2.8^∗^	12.6 ± 2.4^∗∗^	10.7 ± 1.7^∗∗∗^	7.5 ± 0.9^∗∗∗∗^
PON-1 (U/L)	157.4 ± 63.5	140.9 ± 61.3^∗^	110.7 ± 50.3^∗∗^	90.8 ± 43.6^∗∗∗^	59.1 ± 38.5^∗∗∗∗^
eNOS (pg/mL)	59.7 ± 23.5	43.6 ± 20.9^∗^	20.5 ± 18.6^∗∗^	15.3 ± 12.9^∗∗∗^	10.2 ± 8.2^∗∗∗∗^
SDF-1*α* (ng/L)	18.1 ± 4.3	16.3 ± 3.8^∗^	12.0 ± 3.1^∗∗^	9.4 ± 1.7^∗∗∗^	5.9 ± 0.7^∗∗∗∗^

^∗^ *P* < 0.001 (stent length < 12 mm group/stent length 13-26 mm group). ^∗∗^*P* < 0.001 (stent length 13-26 mm group/stent length 30-40 mm group). ^∗∗∗^*P* < 0.001 (stent length 30-40 mm group/stent length 46-56 mm group). ^∗∗∗∗^*P* < 0.001 (stent length 46-56 mm group/stent length>56 mm group).

**Table 5:** Biomarker levels and different reocclusion times in the elderly patients with very long stent implantations.

	Without coronary reocclusion *n* = 61	Coronary reocclusions at 21-25 months *n* = 56	Coronary reocclusions at 16-20 months *n* = 61	Coronary reocclusions at 11-15 months *n* = 65	Coronary reocclusions at 6-10 months *n* = 58
ACR (nmol/mL)	0.9 ± 0.2	2.7 ± 0.6^∗^	5.1 ± 0.4^∗∗^	11.1 ± 2.3^∗∗∗^	15.4 ± 3.1^∗∗∗∗^
MDA (nmol/L)	3.5 ± 1.4	4.8 ± 1.7^∗^	7.2 ± 2.1^∗∗^	12.7 ± 3.5^∗∗∗^	18.0 ± 4.0^∗∗∗∗^
hs-CRP (mg/L)	2.7 ± 0.8	5.8 ± 1.5^∗^	8.1 ± 2.4^∗∗^	13.6 ± 3.7^∗∗∗^	15.9 ± 3.9^∗∗∗∗^
TNF-*α* (ng/L)	20.1 ± 6.2	40.3 ± 6.9^∗^	45.0 ± 7.5^∗∗^	53.8 ± 7.9^∗∗∗^	82.1 ± 9.1^∗∗∗∗^
SOD3 (U/mL)	17.0 ± 3.0	13.2 ± 2.7^∗^	11.3 ± 2.3^∗∗^	7.1 ± 1.2^∗∗∗^	5.0 ± 0.8^∗∗∗∗^
PON-1 (U/L)	157.4 ± 63.5	138.0 ± 60.1^∗^	102.9 ± 56.4^∗∗^	98.2 ± 50.4^∗∗∗^	60.7 ± 41.5^∗∗∗∗^
eNOS (pg/mL)	59.7 ± 23.5	34.2 ± 25.0^∗^	26.3 ± 21.3^∗∗^	12.0 ± 18.5^∗∗∗^	7.9 ± 13.0^∗∗∗∗^
SDF-1*α* (ng/L)	18.1 ± 4.3	15.4 ± 3.8^∗^	10.7 ± 2.6^∗∗^	6.9 ± 1.7^∗∗∗^	4.5 ± 1.1^∗∗∗∗^

^∗^ *P* < 0.001 (without coronary reocclusion group/coronary reocclusions at the 21-25 month group). ^∗∗^*P* < 0.001 (coronary reocclusions at the 21-25 month group/coronary reocclusions at the 16-20 month group). ^∗∗∗^*P* < 0.001 (coronary reocclusions at the 16-20 month group/coronary reocclusions at the 11-15 month group). ^∗∗∗∗^*P* < 0.001 (coronary reocclusions at the 11-15 month group/coronary reocclusions at the 6-10 month group).

**Table 6:** Multiple regression analysis to analyze the statistical significance of variables for complex reocclusion lesions after coronary stenting.

Variables	Odds ratio	95% CI	*P* value
Age	0.59	0.16-3.15	0.12
Gender	2.29	0.47-6.13	0.20
Type II diabetes	1.17	0.35-4.26	0.15
Coronary heart disease	0.30	0.12-1.41	0.37
Hypertension	1.43	0.50-3.17	0.09
Myocardial infarction	2.61	0.49-5.22	0.24
Angina pectoris	0.72	0.26-1.31	0.10
Dyslipidemia	1.80	0.37-7.04	0.43
Married	2.19	0.71-6.25	0.11
Unmarried	0.34	0.19-4.08	0.30
Obesity	1.48	0.42-3.19	0.62
Target lesion revascularization	0.56	0.31-5.23	0.07
Reocclusion lengths	1.46	1.07-3.64	0.01
Lesion calcifications	1.57	1.30-3.59	0.01
Stent lengths	4.31	2.11-6.28	0.002
Reocclusion times	2.36	1.40-3.53	0.01
ACR	3.13	1.23-2.49	0.01
MDA	1.52	1.30-3.07	0.001
Hs-CRP	3.16	2.61-6.25	0.003
TNF-*α*	1.37	1.40-4.74	0.001
SOD3	4.18	2.04-6.28	0.01
PON-1	2.45	1.41-3.16	0.03
eNOS	3.29	2.03-5.39	0.04
SDF-1*α*	1.53	1.36-4.03	0.02

## Data Availability

All relevant data are available within the paper. The data used to support the findings of this study are available from the corresponding author upon request. No additional data are available.
